# Editorial: Role of Coagulation Pathways in Neurological Diseases

**DOI:** 10.3389/fneur.2019.00791

**Published:** 2019-07-23

**Authors:** Svetlana Lorenzano, Matilde Inglese, Tatiana Koudriavtseva

**Affiliations:** ^1^Department of Human Neurosciences, Sapienza University of Rome, Rome, Italy; ^2^Department of Neuroscience, Rehabilitation, Ophthalmology, Genetics, Maternal and Child Health (DINOGMI), University of Genoa, Genoa, Italy; ^3^Ospedale Policlinico San Martino IRCCS, Genoa, Italy; ^4^Neuro-Oncology, Department of Clinical Experimental Oncology, IRCCS Regina Elena National Cancer Institute, IFO, Rome, Italy

**Keywords:** neurological diseases, coagulation, innate immunity, neuroinflammation, neurodegeneration

There is a growing evidence that abnormalities of coagulation pathways are involved in the pathogenesis of several neurological diseases in tight correlation with both neuroinflammation and neurodegeneration. The concept of *thrombo-inflammation* was first introduced in vascular diseases of central nervous system (CNS) (1) closely related to a more general entity of *immunothrombosis*, i.e., formation of thrombi inside microvessels by innate immune cells and specific thrombosis-related molecules, having major physiological role in immune defense rather than in haemostasis (2).

This Research Topic gathers different contributions that added new information on the involvement of both coagulation factors and innate immune components in the pathogenesis of human neurological diseases with the greatest share from the studies on multiple sclerosis (MS) and experimental autoimmune encephalomyelitis (EAE), its animal model.

Plantone et al. focused on the key role of both coagulation and vascular thrombosis in the pathophysiology of MS. The observation of a close concordance between perivascular fibrin(ogen) deposition and the occurrence of clinical signs in EAE has led to numerous studies to investigate the role of thrombin and fibrin(ogen). Most findings supported that blood-brain barrier (BBB) breakdown, presence of active plaques, and disease exacerbation in both humans and animal models are conditions characterized by an increased coagulation activity.

Furthermore, Ziliotto et al. pointed out that increased BBB permeability leads to the irruption into the CNS of blood components including coagulation factors. Their cytotoxic deposition with the activation of microglia, resident innate immune cells, already in pre-demyelinating lesion stage in EAE and MS, cause inflammatory response and immune activation sustaining neurodegenerative events in MS. In particular, among the coagulation factors, FXII could act as an autoimmunity mediator due to its deposition near dentritic cells positive for CD87.

In their research, Ziliotto et al. investigated multiple FXII-related variables, including either its circulating levels, pro-coagulant function, ratio values or variation over time, in 74 MS patients and 49 healthy subjects. They found in MS patients an increased FXII plasma level, a significant difference over time for FXII procoagulant activity and reduced function within the intrinsic coagulation pathway, which supports investigation of FXII contribution to disease phenotype and progression.

Interestingly, the role of the coagulation process entangled with other pathogenic pathways in MS (i.e., a crosstalk between coagulation, inflammation, and immune system), was reinforced by over-connectivity between genome-wide associations MS data and a network of coagulation pathways studied by La Starza et al.. Moreover, genes coding for cluster of differentiation 40 (CD40), especially operative in B lymphocytes, and plasminogen activator urokinase (PLAU) shared both networks, pointing to an integral interplay between coagulation cascade and one of main pathogenic immune effectors.

The involvement of coagulation factors, especially factor XII, fibrinogen and thrombin, beyond their traditional roles in haemostasis, in the development of inflammatory diseases like MS, rheumatoid arthritis and colitis was again the focus of the systematic review by Göbel et al. who highlighted the molecular mechanisms underlying the balance between haemostasis and thrombosis, and between protection from infection and extensive inflammation.

The double nature, thrombotic and immunologic, is also evident in other specific neurologic condition such as the antiphospholipid syndrome (APS) and in the therapeutic strategy adopted for this disorder as discussed in the review by Fleetwood et al.. APS is an autoimmune antibody-mediated condition characterized by thrombotic events and/or pregnancy morbidity in association with persistent positivity to antiphospholipid antibodies. The CNS is frequently affected, as intracranial vessels are the most frequent site of arterial pathology. Nevertheless, ischemic injury is not always sufficient to explain clinical features of the syndrome and immune-mediated damage has been advocated.

Festoff and Citron reviewed available evidence on the role of coagulation cascade activation, in particular of thrombin signaling, in neurodegeneration and in the potential development of effective therapeutic approaches for ALS and traumatic brain injury. Different elements and regulators of the coagulation pathway have significant impact in these conditions and each of these molecules are entangled in choices dependent upon specific signaling pathways in play. For example, the particular cleavage of protease activated receptor 1 (PAR1) by thrombin versus activated protein C will have downstream effects through coupled factors to result in toxicity or neuroprotection.

Thrombin and its PAR1 are potentially important also in peripheral nerve inflammatory diseases as it has been addressed by Shavit-Stein et al. who studied the role of these factors in rat experimental autoimmune neuritis (EAN), a model of the human Guillain-Barre syndrome. The authors showed that thrombin activity in the sciatic nerve was elevated in EAN compared to control sham rats. Furthermore, treatment with non-selective thrombin inhibitors significantly inhibited specific thrombin activity in EAN rats' sciatic and improved clinical scores compared to the untreated EAN rats with normalization of proximal amplitude observed in nerve conduction studies.

The emerging role of coagulation in infectious diseases such as Lyme neuroborreliosis (LB), the most common tick-borne disease involving nervous system caused by the spirochete Borrelia, has been investigated by Di Domenico et al.. In fact, invasive forms of B. burgdorferi are known to expresses multiple plasminogen-binding surface proteins that likely assist pathogen dissemination through host tissues. During the course of the infection, bacteria migrate through the host tissues altering the coagulation and fibrinolysis pathways and the immune response, reaching the CNS within 2 weeks after the bite of an infected tick.

The importance of coagulation system in the management of neurological diseases, particularly in elderly, is also evident by the potential risk associated with the increasing prescription of the new direct oral anticoagulants (DOACs), namely apixaban, dabigatran, edoxaban, and rivaroxaban, in patients with epilepsy taking concomitant antiepileptic drugs (AEDs). As a result, potential interactions may cause an increased risk of DOAC-related bleeding or a reduced antithrombotic efficacy. This issue was evaluated by Galgani et al. who found that there are only few case reports describing such interactions and, therefore, limited evidence is available.

An indirect role of the coagulation system in neurocognitive disorders has been assessed by Alisi et al. who reviewed recent evidence on the emerging involvement of vitamin K, whose biological activity in blood coagulation has been thoroughly explored, even in brain cells development and survival and, hence, in brain functions. In particular, vitamin K seems to have an antiapoptotic and anti-inflammatory effect mediated by the activation of Growth Arrest Specific Gene 6 and Protein S and to be involved in sphingolipids metabolism, a class of lipids that participate in the proliferation, differentiation and survival of brain cells. Vitamin K antagonists, used worldwide as oral anticoagulants, may have a negative influence on cognitive domains such as visual memory, verbal fluency and brain volume.

All these contributions indicate that the study of coagulation pathways in neurological diseases would lead to a greater understanding of their pathophysiology and a more appropriate therapeutic approach. We hope that this Research Topic will help the reader to find a useful reference for the state of the art in this emerging research field and, in particular, both researchers and clinicians to face their challenges with a more complete pathogenic approach since the role of innate immunity and of its effector coagulation factors is very relevant in both health and pathology.

## Author Contributions

SL, MI, and TK all contributed equally to the literature research and writing.

### Conflict of Interest Statement

MI has received research grants from NIH, NMSS, FISM, and Teva Neuroscience; TK has received research grant from the Italian Ministry of Health. The remaining author declares that the research was conducted in the absence of any commercial or financial relationships that could be construed as a potential conflict of interest.
